# Relations in young adults with cancer: acknowledging partners and friends as caregivers—a Danish national study

**DOI:** 10.1007/s00520-026-10952-z

**Published:** 2026-06-30

**Authors:** Marie Ernst Christensen, Helle Enggaard, Helle Pappot, Lise Bjerrum Thisted, Laura Ingrid Braad Carstens, Anne Katrine Hartmann Søby, Tine Lykke, Mathilde Laura Kærgaard Johansen, Mette Buur Bergmann, Maiken Hjerming, Karin Piil, Louise Hennings Jespersen, Anette Borup Simonsen, Elizabeth Rosted, Line Bentsen, - on behalf of the Network for Adolescents Young Adults Cancer Research in Denmark (NAYAcare DK)

**Affiliations:** 1 Research Centre for Prevention and Rehabilitation, Programme for Nutrition & Quality of Life, VIA University College, Aarhus N, Denmark; 2 FOKUS, UCSF - Research and Knowledge Unit for Cancer and Young Adult Health, Copenhagen University Hospital - Rigshospitalet, Section 9701, Ryesgade 27, Copenhagen, Denmark; 3 Clinical Nursing Research Unit & Clinical Cancer Research Center, Aalborg University Hospital, Sdr. Skovvej 15, Aalborg, Denmark; 4 Dept. of Oncology, Centre for Cancer and Organ Diseases, Copenhagen University Hospital, Rigshospitalet, Blegdamsvej 9, Copenhagen, Denmark; 5 Faculty of Health and Medical Sciences, Department of Clinical Medicine, Copenhagen University, Copenhagen, Denmark; 6 Dept. of Clinical Oncology and Palliative Care, Zealand University Hospital, Roskilde, Denmark; 7 University of Copenhagen, Copenhagen, Denmark; 8 Dept. of Oncology, University Hospital of Southern Denmark, Vejle, Denmark; 9 Dept. of Hematology, Cancer Research Center, Aalborg University Hospital, Sdr. Skovvej 15, Aalborg, Denmark; 10 Dept. of Hematology, Zealand University Hospital, Roskilde, Denmark; 11 Dept. of Hematology, Centre for Cancer and Organ Diseases, Copenhagen University Hospital, Rigshospitalet, Blegdamsvej 9, Copenhagen, Denmark; 12 Dept. of Oncology, Centre for Cancer and Organ Diseases, Copenhagen University Hospital, Rigshospitalet, Blegdamsvej 9, Copenhagen, Denmark; 13 School of Nursing, Faculty of Health Sciences, Curtin University, Perth, Western Australia Australia; 14 Youth Cancer, Danish Cancer Society, Strandbouldevarden 49, Copenhagen, Denmark; 15 Dept. of Hematology, Aarhus University Hospital, Aarhus, Denmark; 16 Dept. of Regional Health Research, University of Southern Denmark, Odense, Denmark; 17 Dept. of Oncology, Centre for Cancer and Organ Diseases, Copenhagen University Hospital, Rigshospitalet, Blegdamsvej 9, Copenhagen, Denmark

**Keywords:** Young adults, Cancer, Caregivers, Cancer trajectory, Relational experiences

## Abstract

**Purpose:**

This study explored relational experiences among young adults with cancer and their self-chosen caregivers (friends and partners) across the cancer trajectory.

**Methods:**

A national, multicentre qualitative study was conducted in oncology and haematology settings at all five Danish University Hospitals. Individual semi-structured interviews were undertaken with young adults with cancer (diagnosed when aged 18–39 years) and their self-chosen friends or partners as caregivers. Data were analysed using reflexive thematic analysis and reported in accordance with COREQ.

**Results:**

Nine young adults with cancer (aged 23–36 years) and 13 informal caregivers (aged 22–34 years) participated (partners *n* = 7, friends *n* = 6). Three themes were identified: (1) Holding on to Normality in a Youth Life on Pause, young adult with cancer and caregiver dyads created “breathing spaces” through everyday routines while navigating disruptions in body, mood, and participation in youth contexts; (2) Navigating Help, Needs, and Boundaries, young adults with cancer and their caregiver balanced requesting and offering support, preferred concrete help, and engaged in mutual protection, with professional support rarely accessed; and (3) Shifting Roles in the Relationship, relationships evolved toward “love and logistics”, and support was organised in changing circles of closeness, with friends often occupying an “in-between” position.

**Conclusions:**

Cancer in young adulthood emerges as a relational phenomenon. Acknowledging partners and friends as caregivers, and fostering communication, boundary setting, and role negotiation, may reinforce dyadic coping processes and support the continuity of everyday life.

## Introduction

Young adults, aged 18–39 years, face crucial challenges as they develop their identities, often accompanied by a natural process of detachment from their parents, and begin to build significant relationships in education, career, and romance, and establish their own family life [1–4]. This period of exploration and personal growth is profoundly disrupted by a cancer diagnosis [5]. Cancer adds significant stress, impacting not only health-related quality of life but also interfering with the young adults’ emerging independence and need for support [4, 6].

Like in paediatric oncology, research focus on the impact of cancer on young adults and their familial relationships, particularly highlighting the role of parents as primary caregivers [7, 8]. This emphasis on parents has provided valuable insights into the psychosocial effects of cancer within families. However, this perspective may overlook the broader social support network of young adults, which includes friends and partners [9, 10]. Despite their increasing importance in young adults’ everyday lives, there remains limited knowledge about the role of caregivers such as friends and partners and how they contribute to the provision of emotional and practical support. These relationships are increasingly central to young adults’ lives, offering emotional support and helping maintain their identity and sense of normalcy despite their cancer experience [11].

There is a significant gap in understanding the challenges faced by friends and partners who are key supportive persons for young adults with cancer. For instance, young adult cancer patients and their partners reported difficulties such as changes in communication, disruptions in expected life milestones, and challenges related to intimacy and body image during the cancer trajectory [12, 13]. Moreover, a scoping review on caregiver burden among adolescents and young adults with cancer found that caregivers beyond parents (including partners and friends) are indeed involved [5]. Thereby, caregivers are often treated as a homogeneous group in research, which may blur the distinct challenges faced by different caregiver populations. This limits insights into the unique dynamics of non-parental and non-sibling caregivers. Finally, another scoping review underscored both the value of friends’ support and the challenges they encounter while also emphasising the lack of studies specifically examining friends’ perspectives and needs [13].

To understand the challenges young adult cancer patients and their close caregivers (friends and partners) face, a nuanced exploration is needed. This may inform a more holistic and person-centred care. Thus, this study aimed to explore the relational experiences of young adults with cancer and their self-chosen caregivers throughout the cancer trajectory.

## Materials and methods

### Study design

This study used a qualitative design with individual semi-structured interviews and reflexive thematic analysis involving young adults with cancer and their self-chosen caregivers (hereafter referred only to caregivers) [14–16]. The study adhered to the Consolidated Criteria for Reporting Qualitative Research (COREQ) [17].

### Setting and patient representative involvement

The study was initiated within the national Network for Adolescents and Young Adults Cancer Research in Denmark (NAYACareDK) [18] and informed by experiences from patient representatives in the network, who emphasised the importance of partners and friends during the cancer trajectory while also recognising challenges faced by the caregivers.

This national, multicentre study was conducted in oncology and haematology settings at all five Danish University Hospitals. The project group, formed within NAYACareDK, was multidisciplinary (nurses, physicians, psychologist, nutritionist) with both research and clinical backgrounds. The patient representative contributed to the study design, refinement of interview guides and written materials, and discussions of findings to support relevance and trustworthiness.

### Participants and recruitment

Purposeful sampling was applied [19]. Young adults with cancer were eligible if they at diagnosis were between 18 and 39 years and diagnosed with a haematological or oncological cancer within the past 5 years and at least 6 months prior to recruitment—either in active or palliative treatment (chemotherapy, radiotherapy, immunotherapy, or targeted therapy) or in follow-up. Participants had to be able to read, understand, and speak Danish and provide informed consent and were excluded if they had received surgical treatment only or had a life expectancy of less than 6 months.

The caregivers were identified by the young adults with cancer as significant persons (partner, spouse, or friend) involved in their cancer trajectory. As the study focused on relational experiences, inclusion in the final sample required that the young adult identified at least one eligible caregiver.

They had to be aged ≥ 18 years, able to read, understand, and speak Danish, and provide informed consent. The caregivers were excluded if they were healthcare professionals involved in the patient’s cancer care or family members other than spouses (e.g. parent, sibling, child, other relatives).

The recruitment aim was two young adults with cancer from each university hospital, seeking variation in cancer type (haematological/oncological), age, gender, and geography. This purposive strategy was operationalised through site-based consecutive recruitment of eligible patients during clinical visits while monitoring variation in the overall sample. Clinicians at the hospitals approached eligible patients during visits and provided written and oral study information. Patients who expressed interest were contacted by a researcher from the NAYACareDK project group. Participating young adults with cancer were asked to invite one or two caregivers; if no eligible caregiver was identified or agreed to participate, the young adult would not be included in the final sample.

If a caregiver was interested, the young adult with cancer forwarded the caregiver’s contact details to the researcher, who then provided written and oral information.

### Data collection

Data were collected from March to September 2025 through individual semi-structured interviews at a time and location chosen by participants (hospital office, online, or by telephone). Separate interview guides were developed for young adults with cancer and the caregivers, based on the study aim, existing literature on young adults with cancer and caregiving, and input from the patient representative. The guides were pilot tested with an eligible young adult with previous cancer and an eligible caregiver (not parent or sibling) and subsequently refined for clarity and relevance; for example, probes were added to explore changing support needs, boundaries, and relational roles during the cancer trajectory.

Interviews were conducted by experienced qualitative researchers (LB, TL, AKHS, HE, LBT, MLKJ). Interview questions were flexibly adapted in response to participants’ answers to support a natural conversational flow [17, 20]. Interviews were audio-recorded.

### Data analysis

Interviews were transcribed verbatim by LB, TL, AKHS, HE, LBT, and MLKJ using a shared transcription guide to ensure uniformity [21]. Transcripts were analysed using reflexive thematic analysis inspired by Braun and Clarke’s six steps [14]. The analytic process involved iterative reading, initial coding, development and refinement of themes, and interpretative synthesis.

To capture contextual nuances and strengthen credibility, researchers (LB, KP, MJ, MEC, AKHS, HE, MKJ, LBT, MBB, ER) initially coded transcripts independently within their respective sites. NVivo (version 14) supported data management in three of five university hospitals; the remaining sites managed data manually using annotated transcripts. Preliminary codes were then presented and discussed in the full author group to reconcile interpretations and agree on final themes. To support reflexivity during theme development, the team engaged in ongoing collaborative discussions and peer debriefing. These practices were used to reflect on assumptions, positionalities, and potential biases and to strengthen the credibility and depth of the thematic findings. These discussions were not aimed at achieving inter-coder reliability, but rather at deepening interpretative engagement with the data through reflexive dialogue across disciplinary and clinical perspectives, in line with reflexive thematic analysis [14].

Although the study draws on a dyadic perspective, interviews were conducted individually with young adults with cancer and their self-chosen caregivers. The term “dyadic” is therefore used conceptually to capture relational processes, shared meaning-making, and mutual influence across perspectives, rather than as a formal dyadic analytic design. Analytically, themes were developed across accounts from both parties to illuminate relational dynamics over the cancer trajectory, rather than to compare or match paired interviews.

The researchers’ clinical backgrounds were treated as an analytic resource, informing sensitivity to relational processes in supportive cancer care, while reflexive dialogue was used to interrogate assumptions and maintain analytic openness.

### Ethical considerations

The project was reported to local Data Protection authorities at all five sites, and a national collaboration agreement was signed from all participating sites. Under Danish legislation, the study did not require approval from a scientific ethical committee because it involved interviews without further intervention [22]. In accordance with the Helsinki Declaration [23], all participants received written and oral information about voluntary participation, pseudonymity, and the right to withdraw at any time without consequences for current or future contact with the Danish healthcare system prior to providing written informed consent.

## Results

Interviews were audio-recorded and lasted 31–80 min (mean 56 min). Out of 11 invited young adults with cancer, 9 consented to participate; five young adults with cancer participated with one caregiver and four with two caregivers (each relation defined as dyads). All 13 invited caregivers participated. The ages of the young adults with cancer ranged from 23 to 36 years (mean 29.2), and caregivers’ age ranged from 22 to 34 years (mean 28.7). The caregivers were partners (*n* = 6), friends (*n* = 6), or a spouse (*n* = 1). Participant characteristics are presented in Table 1 (a) (young adults with cancer) and (b) (caregivers).

**Table 1:** (a) Demographic and cancer-related clinical data of the young adults with cancer (*n* = 9). (b) Demographic data of the young adult caregivers (*n* = 13)

(a)	
	***n***
*Gender*	
Female	4
Male	5
*Mean age at interview, y (range)*	29.2 (23–36)
*Ethnic background*	
Danish	9
*Highest educational degree*^*a*^	
Secondary (vocational) education or less	3
University college	3
University	3
*Cancer type*	
Oncological cancer^b^	4
Haematological cancer^c^	5
*Time since diagnosis*	
0–1 year	2
2–5 years	7
*Treatment received*^*d*^	
Chemotherapy	8
Radiation	4
Surgery	4
Bone marrow transplantation	3
Other^e^	4
**(b)**	
	***n***
*Relation*	
Partner/spouse	7
Friend	6
*Gender*	
Female	10
Male	3
*Mean age at interview, y (range)*	28.7 (22–34)
*Ethnic background*	
Danish	13
*Highest educational degree*^*a*^	
Secondary (vocational) education or less	7
University college	3
University	3

^a^The classification is based on International Standard Classification of Education (ISCED 97)

^b^For example, mesothelioma of the tunica vaginalis testis, brain tumour, and breast cancer

^c^For example, leukaemia, and lymphoma

^d^Multiple answers possible

^e^Targeted therapy, antihormonal therapy, and immune therapy

The interviews lasted 31–80 min (mean 56 min). Three overarching themes with associated subthemes were identified: (1) Holding on to Normality in a Youth Life on Pause, (2) Navigating Help, Needs, and Boundaries, and (3) Shifting Roles in the Relationship. The three themes are described below and quotations linked to each theme are presented in Table 2.

**Table 2:** Themes, subthemes, and quotes. Quotes are not presented in dyads

Themes	Subthemes	Quotes
Holding on to Normality in a Youth Life on Pause	Co-constructing normality in a disrupted daily life	Quote 1: “…it was important just to forget about it once in a while, to have that kind of free space” [Female, caregiver, partner]
		Quote 2: “It was hard to strike a balance between doing too much and doing too little” (Female, caregiver, friend)
		Quote 3: “…I was the bald one… something private suddenly became public” [Female, young adult with cancer, dyadic with friends]
		Quote 4: “…he becomes a slightly different person when he has to go for a check-up” [Female, caregiver, partner]
		Quote 5: “…it costs that I work full-time now… I need to relax more, so [my partner] takes the lead on cooking” [Male, young adult with cancer, dyadic with partner]
		Quote 6: “Periods where you have been a little less like a couple and a little more… [practical]… but… we have stood by it” [Female, caregiver, partner]
	Anticipating and fearing the future at the same time	Quote 7: “…I feel there is a before and after… I spend an incredible amount of energy on illness and hospital” [Male, young adult with cancer, dyadic with partner]
		Quote 8: “I have more of the approach that I don’t know if I have tomorrow – so I just need to get everything out of it(…) she [the caregiver] is moving into a more established adult life, whereas I am still more like ‘Okay, now something else needs to happen’” [Female, young adult with cancer, dyadic with friends]
		Quote 9: “Well… I am always worried about how long we will get to have her… I think I try to remember to enjoy it as long as we have each other – to focus on creating good memories and great experiences together” [Female, aregiver, friend]
		Quote 10: “…we quickly agreed that we want to have children… but it is still a concern” [Female, caregiver, partner]
		Quote 11: “…sometimes I have thought that it would be easier if I didn’t have [a partner]…” [Female, young adult with cancer dyadic with partner]
Navigating needs, boundaries, and mutual protection		Quote 12: “It is hard enough to ask for help… when you experience that your request are not met” [Female, young adult with cancer, dyadic with friend]
		Quote 13: “…I can feel a bit like a burden… it is just really hard for me to ask for help… can you make a lasagna?” [Male, young adult with cancer, dyadic with partner]
		Quote 14: “The worst thing people can do is say ‘just let me know’… the best is simply to call and ask: how about Thursday?” [Male, young adult with cancer, dyadic with partner]
		Quote 15: “…it was kind of like a team… everyone sort of stepped up” [Male, young adult with cancer, dyadic with partner]
		Quote 16: “Everyone thinks others are doing something… [it becomes/I felt] lonely” [Female, young adult with cancer, dyadic with friend]
		Quote 17: “Well, you have probably somehow had to put yourself a bit more aside… during the cancer course, you probably put it a bit aside, your own things I mean…” [Female, caregiver, partner]
		Quote 18: “I think it would be somewhat unnecessary for F to have to deal with that (the friends concerns) when she already had enough to think about” [Female, young adult with cancer, dyadic with friends]
		Quote 19: “…bring the good mood along… I am proud of how I have handled the whole process… to be decent towards… family and friends.” [Male, young adult with cancer, dyadic with partner]
		Quote 20: “The fact that she could, as far as possible, still live a life and still do things with friends […] I don’t think that has been negative, but I simply think that is just to take care of oneself.” [Male, young adult with cancer, dyadic with partner]
		Quote 21: “[My partner] also had a hard time letting go of me […] it’s strange, you know, that you’re just left at a hospital. I can just go home and live my life, and [my partner’s] life has just come to a standstill, right?” [Female, YA caregiver, partner]
		Quote 22: “Nothing more than just talking about it with my family… that has also been difficult; many of my closest friends are also colleagues with him, so there had to be a boundary… things that are private… I haven’t sought any help from anywhere.” [Female, YA caregiver, partner]
Shifting Roles in the Relationship	Between love and logistics	Quote 23: “…it was hard to have to sit in a room and be her friend, when I was also the one who was ill with cancer” [Female, YA with cancer, single]
		Quote 24: “…I felt I was protecting them by staying positive and continuing to contribute to everyday life, even if I could not perform an entirely ‘equal’ relationship” [Female, YA with cancer, in relation with partner]
		Quote 25: “She, after all, had to be there after my surgeries, where she has been a caregiver and, yes, a cook and had to do the cleaning, while I have just been lying completely still… she has had to manage everything alone” [Male, YA with cancer, in relation with partner]
		Quote 26: “I am a relative, not a health professional” [RH3a]
		Quote 27: “I can’t have that conversation… to give that awful thought space… feels like the thing that could get me down” (Female, YA caregiver, partner)
	Circles of closeness	Quote 28: “My family… was closer… [but the friend] was also a relative” [Male, YA with cancer, in relation with partner]
		Quote 29: “Now… [she is] coming along to all the check-ups… before, my mother came along a lot” [Male, YA with cancer, in relation with partner]
		Quote 30: “It depends on where you are in the relationship… back then it was most natural that it was my parents who were the closest” [Male, YA with cancer, in relation with partner]
		Quote 31: “You do come from far away, even though the feelings are strong… you are at a distance. I also think that’s how we should be. But get as close as possible…” [Female, YA caregiver, friend]
		Quote 32: “Hard to find that balance between how much and how little one could do” [Female, YA caregiver, friend]

### Holding on to normality in a youth life on pause

Normality was continuously redefined in everyday interactions and renegotiated as roles and perspectives shifted. While caregivers broadly shared a commitment to “keeping things normal”, partners and friends differed in expectations and ways of being involved.

#### Co-constructing normality in a disrupted daily life

Across the relationship between the young adults with cancer and their caregiver, ordinary routines were used to create “breathing spaces” where cancer did not define daily life. Walks, shared meals, TV series, and seeing friends helped affirm the young adults with cancer as more than a patient. Young adults with cancer described relief in being treated as a young person with everyday concerns, while caregivers described deliberate efforts to sustain positivity and protect the relationship from being overshadowed by illness (Table 2, quote 1).

A difference appeared among partner caregivers versus friend caregivers. Partners often experienced an expectation, also from family and healthcare professionals, of continuous hands-on involvement. They assumed logistical and practical tasks while trying to preserve a sense of “us”. Friends typically provided more episodic support, reintroducing pre-illness activities and social outings without daily caregiving obligations. Friends experienced the ongoing puzzle of wanting to help without overstepping (Table 2, quote 2).

Normality was repeatedly challenged by physical, emotional, and social changes. Bodily changes (e.g. hair loss, weight changes, fatigue) made the private visible and complicated self-presentation in youth contexts such as study, work, and nightlife (Table 2, quote 3). Young adults with cancer described feeling exposed, while the caregivers adjusted to fluctuating mood and energy, particularly around follow-up cycles (Table 2, quote 4).

Work and study were central markers of “getting back”, but returning also required redistribution of everyday responsibilities. Resuming full-time work could signal moving forward, yet practical burdens often shifted: partners more frequently took over household tasks, whereas friends maintained social connection and discretion without requiring disclosure (Table 2, quote 5). Young adults described the “double work” of being both “the sick one” and a whole person in youth settings, sometimes performing strength to protect others, sometimes withdrawing to manage exposure. Partners described arriving at a “new normal” after periods of feeling “less partner, more organiser”, before gradually finding their way back to couple hood (Table 2, quote 6).

#### Anticipating and fearing the future at the same time

Looking beyond treatment, futures were drafted and redrafted. Young adults with cancer described a lasting “before and after” shaping decisions about study, work, holidays, and moving. Follow-up appointments reintroduced uncertainty, and late effects or fluctuating energy required contingency. Partners more often anticipated shared timelines and joint decision-making, which intensified the weight of planning, while friends tended to support belonging and near-term goals without expectations of long-term life plans (Table 2, quote 7).

For some young adults with cancer, living more in the present became a way of coping, while partners and friends often remained oriented toward established adult aspirations (Table 2, quote 8). Friends also described the future as containing both worry and hope, making it easier to focus on the present rather than plan too far ahead (Table 2, quote 9).

Fertility concerns emerged as part of future-oriented thinking. Some dyads described sustained wishes for children alongside uncertainty about recurrence and treatment effects. Partners sometimes held worries back to protect the other, while young adults with cancer at times postponed fertility conversations to avoid straining the relationship (Table 2, quote 10). In one case, ambivalence about the future was intensified by a newer relationship; imagining solitude appeared as a seemingly simpler alternative, reflecting the strain of both needing support and not wanting to burden the partner (Table 2, quote 11).

### Navigating needs, boundaries, and mutual protection

Across relationships, the dyads described ongoing negotiation between expressing needs, setting boundaries, and protecting each other. Asking for help could feel exposing and burdensome, especially when requests were unmet (Table 2, quote 12). Young adults with cancer found concrete, proactive offers easier to accept than open-ended invitations such as “let me know if I can help” (Table 2, quotes 13–14). Informal caregivers were strongly motivated to assist, yet uncertainty about when and how to act could make even well-intended support feel pressuring. Friends more often set limits, for example, by not attending hospital appointments, whereas partners typically provided daily practical and logistical support.

Practical needs were most pronounced when the young adults with cancer had low energy, but these needs often eased over time. Several participants described a “team” approach, in which tasks were distributed among partners and family members to avoid overburdening a single caregiver (Table 2, quote 15). However, when responsibilities were assumed to be covered by “someone else”, support could fall through (Table 2, quote 16).

Mutual protection frequently meant down-prioritising one’s own needs. Some caregivers described setting aside their well-being to support the young adults with cancer, which felt understandable but difficult to sustain alongside work, family, and household duties (Table 2, quote 17). Similarly, young adults with cancer described trying to protect their caregivers by maintaining morale and consideration even when depleted. Both sides regulated emotions and effort to avoid overburdening the other; this was often experienced as protective but could be emotionally taxing and sometimes create distance (Table 2, quotes 18–19). Some caregivers also described needing boundaries to protect their own well-being while staying present (Table 2, quote 20). During hospital stays, periods of separation could be distressing for both young adults with cancer and partner, highlighting tensions between caregiving demands and everyday life (Table 2, quote 21). Caregivers primarily sought support from family or friends to release frustration without burdening the young adults with cancer; professional or organisational support was rarely used (Table 2, quote 22).

### Shifting roles in the relationship

Across the cancer trajectory, roles shifted in ways that could feel meaningful yet challenging in the context of young adult life.

#### Between love and logistics

The dyads described how the original relationship role (partner/close friend) became insufficient as care responsibilities expanded. Young adults with cancer noted a visible shift from being “the friend” to becoming “the one with cancer”, making ordinary social situations harder to navigate (Table 2, quote 23). Some consciously downplayed symptoms and performed strength to protect partners and friends from the full weight of illness (Table 2, quote 24).

Partners described how partnership increasingly merged with practical caregiving: cooking, cleaning, medication management, coordinating hospital appointments, and providing care (Table 2, quote 25). Some described being “used as a nurse” for wound care, which prompted deliberate reminders of relational boundaries (Table 2, quote 26). When one person became the primary “fixer”, caregivers could feel isolated and sometimes set emotional limits, for example, by not absorbing every existential worry (Table 2, quote 27).

#### Circles of closeness

Support was typically organised as a layered and shifting network around the young adults with cancer. Partners were often positioned in the innermost circle and expected to attend hospital visits and participate in decisions, while friends tended to occupy a supportive role with fewer obligations (Table 2, quote 28). In some dyads, a friend moved into the inner circle, particularly when a friendship developed into a romantic relationship during the trajectory and could begin joining consultations and sometimes “take over” from a parent (Table 2, quote 29). Placement also depended on the maturity of the couple relationship; newer relationships did not automatically supersede parental involvement (Table 2, quote 30). Friends often supported from a respectful distance, offering emotional support, distraction, and everyday normality while deferring to parents or partners, creating an “in-between” position of being “very close and yet far away” (Table 2, quotes 31–32). From this position, friends continually balanced being present with not overstepping the inner circle.

## Discussion

This study illustrates how young adults with cancer and their self-chosen caregivers jointly navigate cancer during a formative life phase. When cancer interrupts a life phase characterised by identity formation and relational change, both parties may face responsibilities they feel only partially prepared for. Across dyads, our findings point to a shared cancer trajectory of holding on to normality in a changed daily life. Creating “breathing spaces” through ordinary routines helped keep the relationship anchored in everyday youth life rather than being dominated by illness, supporting previous findings [12].

Efforts to maintain normality were repeatedly tested by treatment consequences, fluctuating energy, and shifting participation in youth contexts. Returning to work or study was experienced as both a marker of moving forward and a trigger for redistributing practical responsibilities in the network, typically with partners absorbing day-to-day tasks while friends maintained connection and discretion. This duality resonates with previous studies documenting high rates of return to work among young adult cancer survivors alongside reduced hours, role renegotiations, and financial strain; it also aligns with emerging survivorship literature framing disability–related barriers and accommodations as part of young adults with cancer post-treatment identity work [24–26].

Looking beyond treatment, both young adults with cancer and their caregivers described futures that were continually revised. Follow-up appointments and concerns about late effects meant that uncertainty persisted beyond treatment, rendering future planning tentative rather than stable. This resonates with survivorship literature describing ongoing health-related risk and fragmented follow-up in AYAs with cancer, and the resulting need for structured, developmentally appropriate survivorship care [27, 28]. Dyads also navigated different temporalities: some young adults with cancer leaned toward living in the present, whereas partners often oriented to longer-term couple timelines. Previous research indicates that future-oriented concerns, such as fear of recurrence, fertility, and financial insecurity, may conflict with a more present-focused coping approach, creating ambivalence and misalignment within close relationships [29]. Fertility concerns were emblematic of this future work: several dyads postponed or protected fertility conversations to avoid adding strain, mirroring qualitative studies where AYAs describe hope for future parenthood amid rushed decisions at diagnosis and ongoing uncertainty about reproductive potential [30–32].

Another key finding concerns the ongoing negotiation of help, needs, and boundaries. Young adults with cancer expressed ambivalence about asking for support and fears of burdening others, while the caregivers described uncertainty about when and how to step in. Our findings align with dyadic coping research and meta-analytic evidence: supportive dyadic coping is linked with better emotional functioning and relationship quality, whereas negative dyadic coping and protective buffering (hiding one’s worries to shield the other) are associated with poorer adjustment [6, 33].

A previous study described how caregivers often found it emotionally difficult to step back for work, study, or everyday obligations. Although the practical necessity was generally acknowledged, doing so could feel painful, especially when it underscored the contrast between a life “on pause” and a life that continues [34]. This mirrors our findings where caregivers primarily sought support from family or friends, while professional or organisational support was rarely accessed, consistent with reports of limited formal support options for young adults with cancer and their caregivers [35, 36].

Finally, our findings show how changed needs were embedded in shifting relational roles and circles of closeness. Partners were often positioned in the innermost circle with expectations of near-constant availability and involvement, while friends occupied a supportive role that was emotionally close yet often more flexible. Friends described an “in-between” position, close but holding back when parents or partners were present, reflecting nuanced relational work across the trajectory. Our results also suggest that newer romantic relationships may coexist with strong parental involvement, complicating expectations about who is “closest” and who takes on which tasks. These shifting roles are not only burdensome; they also highlight the capacity for support and resilience within young adult relationships [9, 10].

Future research should further examine how partners, friends, and parents are positioned differently within young adults’ changing circles of closeness, and how developmentally appropriate interventions may support communication, boundary setting, and role negotiation across the cancer trajectory. Clinically, our findings together with others suggest that healthcare professionals should recognise and, where relevant, engage the broader social network around young adults with cancer, validating both partners and friends as important caregivers and attending to the relational work required to keep relationships from being reduced to care alone [37, 38]. Supporting clarity around boundaries, practical coordination, and communication may help transform role shifts from hidden strain into a shared resource for coping [39].

In practice, this may involve explicitly asking young adults with cancer whom they consider significant supporters, inviting partners or friends into consultations when appropriate, and briefly addressing how support is organised and negotiated in everyday life. Simple, structured questions about boundaries, unmet needs, and role expectations may help identify relational strain early, particularly as professional support was rarely accessed despite substantial caregiving demands.

### Strengths and limitations

This national, multicentre qualitative study included participants across Denmark’s university hospitals, supporting variation and transferability [40]. A strength was the collaboration with a patient representative in the development of the study and that the interview guide was pilot tested with a young adult cancer survivor and a self-chosen caregiver. Also strengthening the study was that the data collection and reporting followed COREQ-32, and analysis involved a multidisciplinary author group.

Limitations include the involvement of five interviewers, which may have introduced variation in interviewing style. However, shared preparation and the use of a common interview guide helped promote consistency across interviews. To ensure pseudonymity, findings were not presented by hospital, diagnosis, or treatment context, limiting contextual detail. In addition, the involvement of multiple researchers may have led to an emphasis on overarching patterns at the expense of finer nuances, despite iterative discussions throughout the analytic process.

## Conclusion

Young adults with cancer and their self-chosen caregivers described cancer as a shared relational experience occurring during a life phase normally characterised by increasing independence, identity formation, education, work, friendship, intimacy, and future planning. Across dyads, cancer placed youth life on pause and required partners and friends to move between ordinary relationship roles and caregiving responsibilities.

The findings show how young adults with cancer and their caregivers worked to preserve normality through everyday “breathing spaces”, negotiated help, needs, and boundaries, and adapted to shifting roles within changing circles of closeness. These processes were shaped by AYA-specific concerns, including disrupted participation in study, work, and social life, uncertainty about the future and fertility, ongoing parental involvement, and friends’ often “in-between” position as emotionally close but not always recognised as caregivers.

Clinically, the findings underline the importance of asking young adults whom they consider significant supporters and of recognising partners and friends as part of the caregiving network while supporting communication, boundary setting, and role negotiation in ways that acknowledge the developmental context of young adulthood.

## Data Availability

The data generated and analysed in this study is not publicly available due to the Danish data protection rules but could be available from the corresponding author on reasonable request.
